# Sixty years of environmental change in the world's largest freshwater lake – Lake Baikal, Siberia

**DOI:** 10.1111/j.1365-2486.2008.01616.x

**Published:** 2008-08

**Authors:** STEPHANIE E HAMPTON, LYUBOV R IZMEST'EVA, MARIANNE V MOORE, STEPHEN L KATZ, BRIAN DENNIS, EUGENE A SILOW

**Affiliations:** * National Center for Ecological Analysis & Synthesis, University of California – Santa BarbaraSanta Barbara, CA 93101, USA; † Scientific Research Institute of Biology, Irkutsk State UniversityIrkutsk 664003, Russia; ‡ Department of Biological Sciences, Wellesley CollegeWellesley, MA 02481, USA; § Northwest Fisheries Science CenterNOAA Fisheries, Seattle, WA 98112, USA; ¶ Departments of Statistics and Fish and Wildlife Resources, University of IdahoMoscow, ID 83844, USA

**Keywords:** *Daphnia*, global warming, great lakes, high-latitude, long-term ecological research, plankton, Russia, subarctic, time series, trophic structure

## Abstract

High-resolution data collected over the past 60 years by a single family of Siberian scientists on Lake Baikal reveal significant warming of surface waters and long-term changes in the basal food web of the world's largest, most ancient lake. Attaining depths over 1.6 km, Lake Baikal is the deepest and most voluminous of the world's great lakes. Increases in average water temperature (1.21 °C since 1946), chlorophyll *a* (300% since 1979), and an influential group of zooplankton grazers (335% increase in cladocerans since 1946) may have important implications for nutrient cycling and food web dynamics. Results from multivariate autoregressive (MAR) modeling suggest that cladocerans increased strongly in response to temperature but not to algal biomass, and cladocerans depressed some algal resources without observable fertilization effects. Changes in Lake Baikal are particularly significant as an integrated signal of long-term regional warming, because this lake is expected to be among those most resistant to climate change due to its tremendous volume. These findings highlight the importance of accessible, long-term monitoring data for understanding ecosystem response to large-scale stressors such as climate change.

## Introduction

Anthropogenic climate change has raised air and water temperatures worldwide (Houghton *et al.*, 2001), and environmental warming in Siberia has surpassed estimates of warming elsewhere (Serreze *et al.*, 2000; Shimaraev *et al.*, 2002;). For high latitude locations that now evidence pronounced temperature increases, the corresponding biological change on land has been significantly greater than that for lower latitudes (Root *et al.*, 2003). However, observed biological responses to climate change within fresh water systems at contrasting latitudes are fewer in number, and seemingly quite heterogeneous [e.g. (Quayle *et al.*, 2002; O'Reilly *et al.*, 2003;)]. Here, we capitalize on a virtually unknown long-term dataset, and describe significant physical and biological changes occurring in Lake Baikal during the past 60 years. These results show how the world's largest lake – a system among those most resistant to climate change and other stressors because of its enormous volume and thermal inertia – is now changing, and they demonstrate the critical role that long-term research and monitoring play in alerting the scientific and management community to ecosystem change.

Spanning more than 4° of latitude and obtaining a maximum depth greater than 1.6 km, Siberia's Lake Baikal is the world's deepest, largest (by volume), and most ancient lake. Because of its tremendous size, it influences climate in a region that is experiencing dramatic environmental warming (Serreze *et al.*, 2000; Shimaraev *et al.*, 2002; Smith *et al.*, 2005; Walter *et al.*, 2006;). Endemism and biotic diversity of this ancient lake is notably high, contributing to UNESCO's 1996 decision to designate Lake Baikal a World Heritage Site. The unusual endemic fauna include the world's only freshwater pinniped (the Baikal seal *Phoca sibirica*), 344 species of amphipods – several of which exhibit gigantism (e.g. *Acanthogammarus maximus*) – and 33 species of sculpin fishes, including the deep-dwelling translucent golomyanka (*Comephorus baicalensis* and *Comephorus dybowskii*) that resemble abyssal marine fishes.

On Lake Baikal, since 1945, three generations of biologists in a single Siberian family have quietly maintained one of the world's most detailed and consistent limnological monitoring programs. Collected at intervals of 7–10 days through all seasons of the year, the physical and biological data are of inestimable scientific importance, spanning 60 years of ecological change at high temporal and taxonomic resolution. These data reveal significant increases in water temperature, consistent with recent reports of rapid Siberian warming (Serreze *et al.*, 2000; Shimaraev *et al.*, 2002;) and long-term changes in Lake Baikal's ice cover (Magnuson *et al.*, 2000; Todd & MacKay, 2003;). In addition, we report significant changes in algal biomass and zooplankton composition that have implications for nutrient cycling in this ultra-oligotrophic system.

## Methods

**Fig. 1:**
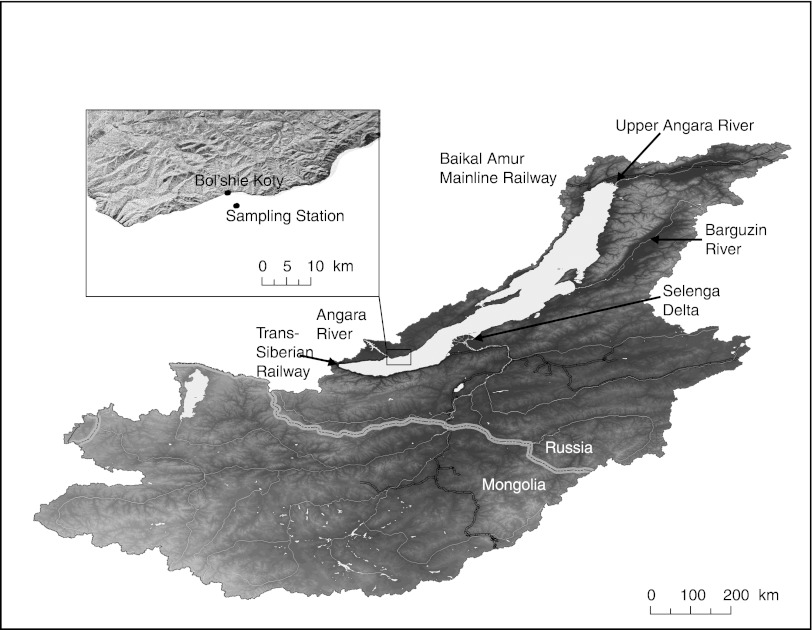
Lake Baikal and its watershed (Heim *et al.*, 2008), showing the Russian–Mongolian border, railways, major inflows (Selenga River, Upper Angara River, and the Barguzin River) and the primary outlet, the Angara River. The sampling station from which data were analyzed is 2.7 km offshore from Bol'shie Koty (51.9018, 105.0665) over water approximately 800 m deep.

Since 1945, data have been collected at least monthly, generally every 7–10 days, in depth profiles from the surface to 250 m at a single main station in the southern basin approximately 2.7 km offshore from Bol'shie Koty where water depth is approximately 800 m (Fig. 1). This station is not influenced by discharge from the Baikalsk pulp mill, located more than 80 km to the south (Kozhova & Izmest'eva, 1998; Mackay *et al.*, 1998;). Sixty-nine other stations throughout Lake Baikal are surveyed once a year, although those data are not presented here. Thin ice prohibits collection in some months, usually January. Water temperature is measured using a mercury thermometer in samples retrieved on deck with a Van Dorn bottle. Secchi depth, the depth at which a standard white disk disappears from view in the water column, is routinely measured as an index of water quality. Zooplankton and phytoplankton samples are enumerated at the species level and zooplankton are also identified by age class. Discrete depths of 0, 5, 10, 25, 50, 100, 150, 200, and 250 m are targeted for measurement of abiotic variables and sampling of phytoplankton with a 10 L Van Dorn bottle. Single zooplankton samples are collected with a closing plankton net (37.5-cm diameter, 100-μm mesh) from depth layers of 0–10, 10–25, 25–50, 50–100, 100–150, 150–250, and 250–500 m. Zooplankton samples have been fixed and stored in formalin throughout the long-term monitoring program; until 1973, phytoplankton samples were also fixed in formalin, but now are fixed with Lugol's solution. In addition, chlorophyll *a* has been measured since 1979 as a proxy for phytoplankton biomass, using standard acetone extraction and spectrophotometry (UNESCO, 1966). For analyses, here we have used temperatures at 0, 25, and 50 m. For biological data, we averaged data within the top 50 m of the lake, the portion of the water column containing most of the lake's photosynthetic production, as well as the summer thermocline (Kozhova & Izmest'eva, 1998). To examine long-term trends for Lake Baikal temperature and zooplankton, we averaged data by quarters to create winter, spring, summer, and fall values. For chlorophyll *a*, only averages of July through August were analyzed here to minimize missing values – the chlorophyll *a* data have only one missing value in these months – and we have verified that trends are strong and consistent when more or fewer months are included in analyses. The chlorophyll *a* trends were compared with trends in July–August averages of Secchi depth. Untransformed data are available through the NCEAS Public Data Repository (Izmest'eva, 2006).

### Long-term trend analysis

We have extracted the major trends in temperature, phytoplankton, and zooplankton to determine whether Lake Baikal has exhibited responses that are consistent with recent evidence of warming in aquatic systems worldwide (Magnuson *et al.*, 2000; Quayle *et al.*, 2002; O'Reilly *et al.*, 2003; Verburg *et al.*, 2003;). The time series of quarterly temperature and log-transformed zooplankton data were nonstationary, with both the magnitude and phase of the annual seasonality varying across the 60-year series. To discriminate the long-term trend from the annual seasonality, the data were subjected to a Discrete Short-Time Fourier Transform (DSTFT) on overlapping 10-year segments of the time series (Qian & Chen, 1996). The frequency corresponding to the annual seasonality in the 10-year segment was set equal to the mean noise level, and the data were then inverse-transformed back to the time domain. The filtered, overlapping, discrete 10-year output time series were reassembled into a continuous 60-year, deseasoned time series using the ‘overlap-save’ method (Press *et al.*, 1994). Frequency domain deseasoning and time-frequency analysis were performed with labview Software (Ver. 8.0, National Instruments, Austin, TX, USA). We used linear regression to determine whether long-term trends were evident across years in the deseasoned data. In all cases, we tested the regression residuals for autocorrelation using statgraphics+ (Ver 3.0, Statistical Graphics, Rockville, MD, USA) before proceeding with analyses. Deseasoning of the time series data successfully removed significant first-order autocorrelation in the residuals in all time series except that of the relatively long-lived copepods (∼2 years lifespan). For the copepod data, we used linear regression with an explicit estimator for first-order autocorrelation of errors (proc autoreg, sas 9.1, SAS Institute). Where trends existed in complete datasets, we also examined the presence of trends within each of the four quarters by sorting the complete data by quarters and performing regression on each of the four resulting datasets. For annual summer means of Secchi depth and chlorophyll *a*, we used simple linear regression after Durbin–Watson tests showed no evident interannual autocorrelation.

### Analysis of plankton–environment relationships

To identify potential drivers of plankton dynamics, we subjected monthly data from 1974 through 1997 to multivariate autoregressive (MAR) analysis (Ives *et al.*, 2003; Hampton & Schindler, 2006;). These years were chosen to minimize missing values (zooplankton missing dates=4, phytoplankton missing dates=23, total dates=264). For algal groups, the change in preservation techniques for the phytoplankton samples in 1973 complicates our ability to examine the full time series in a single analysis. We grouped plankton into nine broad taxonomic categories: copepods, rotifers, cladocerans, chrysophytes, cryptophytes, cyanobacteria, diatoms, dinoflagellates, and green algae. Where data were missing, we used linearly interpolated values. In the case of the phytoplankton, no 1991 data were available – for these samples, we substituted average values for each month. The data series were ln-transformed, such that the models could characterize nonlinear relationships more effectively (Ives, 1995). Data were standardized to dimensionless units (*Z*-scores), by subtracting the mean for each taxon and dividing by its standard deviation, so that effects in the resulting models were directly comparable.

A MAR model is essentially a set of multiple regressions solved simultaneously for interacting taxa that may both affect and respond to other groups, with time lags between drivers (e.g. abundances of other taxa, values of environmental variables) and responses that explicitly account for first-order autocorrelation in time series. As with other regression techniques, a MAR model reveals relationships that best predict the abundance of each taxon, given the data in hand. The MAR models have been described in detail elsewhere (Ives, 1995; Ives *et al.*, 1999, 2003; Hampton *et al.*, 2006; Hampton & Schindler, 2006). matlab (The Mathworks Inc., Natick, MA, USA) code for MAR models has been made publicly available by Ives *et al.* (2003) through Ecological Archives. The MAR modeling tools are also publicly available as a free executable, the lambda package (http://www.nwfsc.noaa.gov/research/divisions/cbd/mathbio/products_page.cfm). For each of the *i* (1.9) endogenous effects (plankton groups) and *k* (1.5) exogenous effects, we fit the autoregressive model 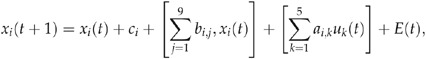
where *x*_*i*_ is the *Z*-score of each taxonomic group [ln(count+1)] at time *t*, *u*_*k*_(*t*) the value of the exogenous variable *k* in units of standard deviation at time *t*, *c*_*i*_ a species-specific constant, and *a*_*i,k*_ and *b*_*i,j*_ are parameters that measure the strength and direction of *k* exogenous and *j* (1...9, including *i*=*j*) endogenous relationships, respectively, for each species, *i*.

Exogenous effects in the model were water temperature (average 0–50 m), month of year, snow depth at the city of Irkutsk, the Arctic Oscillation (AO) index, and the El Niño Southern Oscillation (ENSO). Snow on ice strongly affects the light environment for algal growth (Mackay *et al.*, 2006); we were able to obtain snow depth data at an Irkutsk station from the Historical Soviet Snow Depth dataset (Armstrong, 2001). The Irkutsk station was the available location in closest proximity to the main Baikal limnological station. While snow conditions on the lake likely differ from conditions in Irkutsk, we expected that conditions at Irkutsk would correlate positively with those at the lake and thus provide an index of Baikal snow conditions. The AO and ENSO indices were obtained from the Joint Institute for the Study of the Atmosphere and Ocean (University of Washington, Seattle, WA, USA). Climatic indices, such as AO and ENSO, may usefully ‘package’ weather effects (Stenseth & Mysterud, 2005) in a manner not otherwise reflected in available Baikal data. Common climatic indices such as AO, ENSO, and the North Atlantic Oscillation (NAO) strongly relate with air temperature anomalies at the lake (S. L. Katz & S. E. Hampton, unpublished data), with AO in particular appearing to relate to Lake Baikal ice dynamics (Todd & Mackay, 2003). These interactions are presently under investigation.

To reduce the probability of overparameterization of the model, we restricted interactions to those that were biologically meaningful (Ives *et al.*, 1999). Specifically, we did not allow positive effects of phytoplankton groups on each other nor the effects of snow cover on zooplankton groups. While indirect interactions could lead to such excluded results, we assumed that such effects would be comparatively minimal at the time intervals considered here.

We used Akaike's Information Criterion (AIC) to select the most parsimonious model. To arrive at the best model structure, we randomly constructed 100 model structures by including or excluding coefficients with equal probability, and chose the resulting model with the lowest AIC. The process was repeated 100 times (Ives *et al.*, 1999), resulting in a single model structure with the lowest AIC, from 10 000 random models. This number of iterations has proven robust, compared with more computationally intensive model runs (S. E. Hampton, unpublished data). Coefficients that were retained in less than 15% of the models were dropped (Ives *et al.*, 1999). We then used bootstrapping (*n*=500) of the final model to obtain 95% confidence intervals for the coefficients in the best-fit model. Coefficients with confidence intervals that overlapped zero were eliminated from the final best-fit model (Hampton *et al.*, 2006; Hampton & Schindler, 2006;).

## Results and discussion

**Fig. 2:**
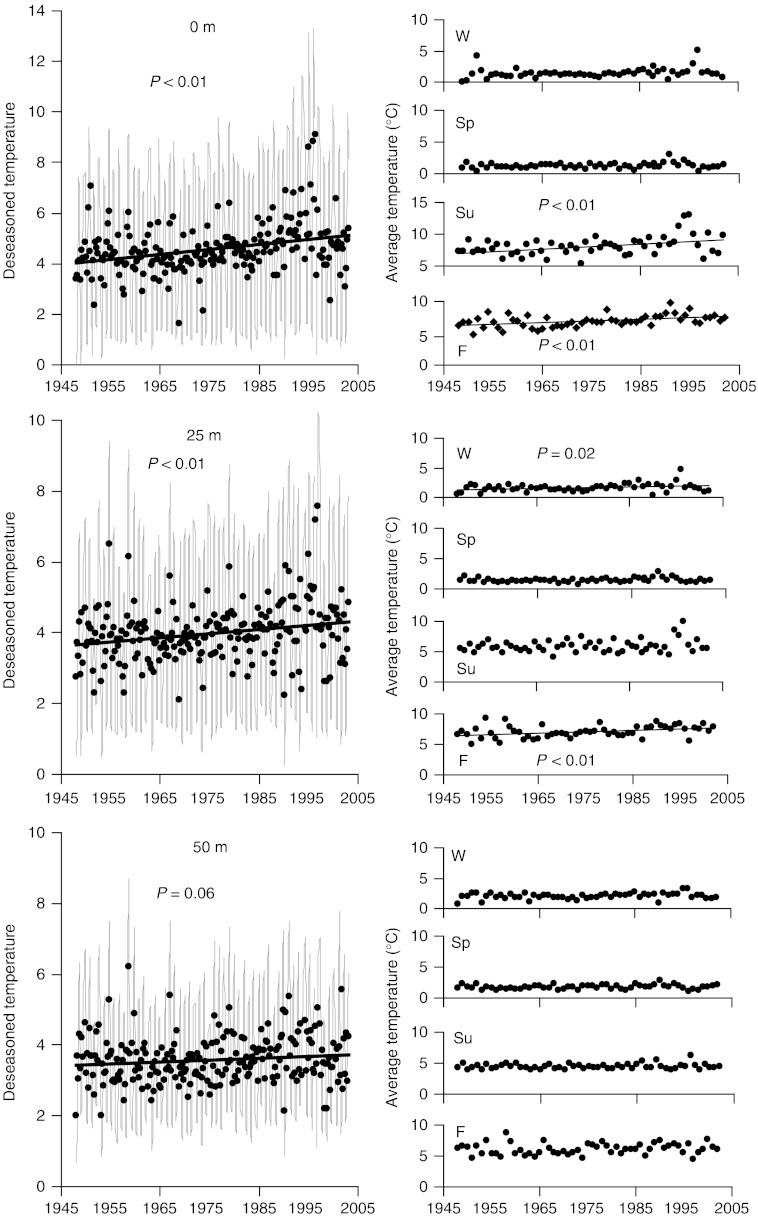
Average quarterly temperature (grey lines) and Discrete Short-Time Fourier Transform ‘deseasoned’ temperature (°C) data at depths 0, 25, and 50 m over 60 years in Lake Baikal. Temperature is segregated by quarter in the right column. Winter (December previous year, January, February) and spring (March, April, May) were typically ice-covered. Summer (June, July, August) and fall (September, October, November) have had incomplete or no ice cover over this time period. Linear regression was used to determine if long-term trends existed, after tests for autocorrelation within the deseasoned and detrended residuals. Significant linear trends were evident for 0 m waters overall (slope=0.02, *R*^2^=0.11, *P*<0.01), and the 25 m depth overall (slope=0.01, *R*^2^=0.06, *P*<0.01). Within quarters, 0 m waters warmed significantly in summer (slope=0.04, *R*^2^=0.15, *P*<0.01) and fall (slope=0.03, *R*^2^=0.20, *P*<0.01), and the 25 m depth warmed in fall (slope=0.02, *R*^2^=0.12, *P*<0.01) and winter (m=0.01, *R*^2^=0.09, *P*=0.02).

**Fig. 3:**
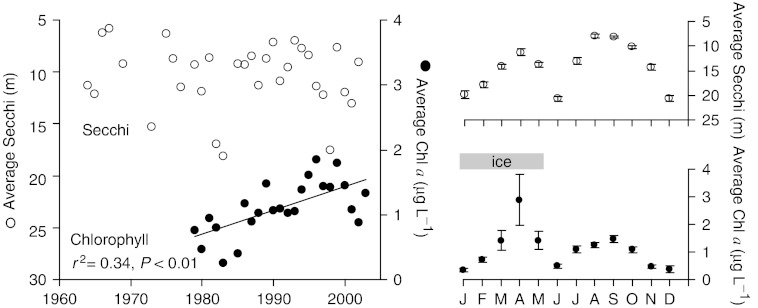
Average, untransformed Secchi depth (open circles, *n*=33) and average chlorophyll *a* content (closed circles, *n*=24; slope=0.04, *R*^2^=0.34, *P*<0.01) for July–August over the full time series, and monthly averages (±1 SE) for both variables. Secchi measurements were available from 1964 to 2002, and chlorophyll *a* measurements were available from 1979 to 2003. Positive trends in chlorophyll *a* were also significant when averaging a greater number of months, but averaging the July–August greatly reduces the number of missing data points. Each variable was analyzed separately with linear regression following nonsignificant results (*P*>0.15) of Durbin–Watson tests for autocorrelation. No trend was apparent in average July–August Secchi depth (*P*=0.58), shown here, nor for Secchi depth within the whole ice-free season (July–November, *P*=0.08). The overall increase in chlorophyll *a* is attributable to both spring and summer increases [nested anova for the 0–50 m stratum of Lake Baikal; year (month) effects on chlorophyll *a*: year (April), year (July), year (August), *P*<0.05].

**Fig. 4:**
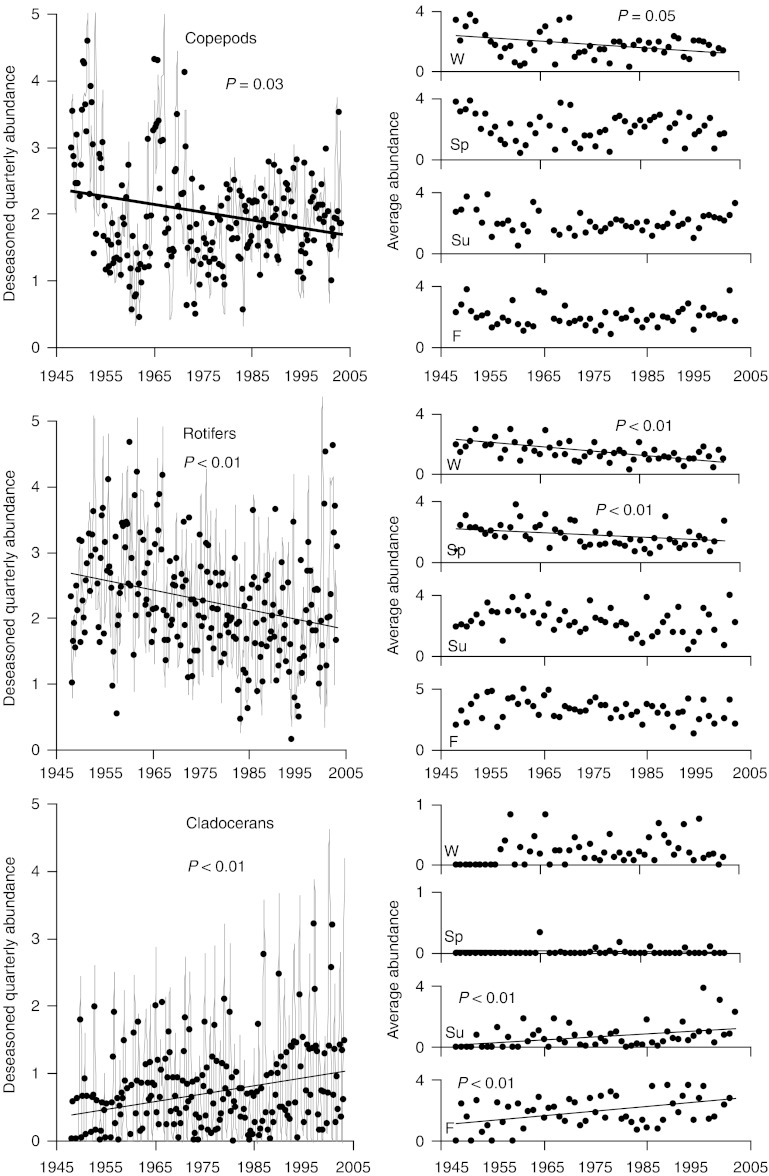
Average quarterly zooplankton abundance (grey lines) and Discrete Short-Time Fourier Transform deseasoned, log-transformed zooplankton abundance [ln(individuals L^−1^+1)], with abundance segregated by quarter in the right column. Winter (December previous year, January, February) and spring (March, April, May) were typically ice-covered. Summer (June, July, August) and fall (September, October, November) have had incomplete or no ice cover over this time period. Linear regression was used to detect long-term trends, after tests for autocorrelation within deseasoned and detrended residuals. Copepod abundances were significantly autocorrelated before and after deseasoning and detrending, so these data were analyzed using a regression model with first-order autocorrelation of errors. Using all data, significant decreases were evident for copepods (slope=−0.01, *R*^2^=0.22, *P*=0.03) and rotifers (slope=−0.02, *R*^2^=0.07, *P*<0.01); cladocerans significantly increased overall (slope=0.01, *R*^2^=0.08, *P*<0.01). Within quarters, copepods decreased in winter (slope=−0.02, *R*^2^=0.18, *P*=0.05), rotifers decreased in winter (slope=−0.03, *R*^2^=0.24, *P*<0.01) and spring (slope=−0.01, *R*^2^=0.09, *P*=0.03), and cladocerans increased in summer (slope=0.02, *R*^2^=0.17, *P*<0.01) and fall (slope=0.03, *R*^2^=0.21, *P*<0.01).

**Fig. 5:**
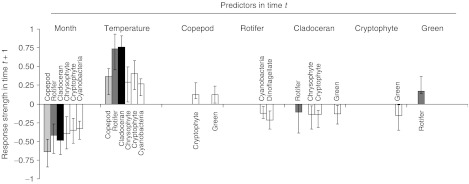
Coefficients, with 95% confidence intervals, resulting from a multivariate autoregressive (MAR) model of Lake Baikal plankton and potential abiotic drivers and climatic indices. The best-fit model was selected from a field of randomly generated models predicting change in taxonomic groups from one time step to the next, with taxonomic groups and exogenous (abiotic) variables in time (*t*) used as predictors for endogenous variables in time *t+*1. Coefficients shown indicate that predictors were significantly positively or negatively related to response variables in the next time step. Bootstrapping of coefficient values in the best-fit model eliminated variables that had confidence intervals overlapping zero. Data were standardized with *Z*-scores such that coefficients are comparable across variables. The variables Arctic Oscillation and snow depth were not retained in the final model and are not shown here. The El Niño Southern Oscillation index was significantly related only to cryptophytes (*a*=0.118), and is not shown.

Overall, we found that Lake Baikal has warmed significantly (Fig. 2), chlorophyll has increased significantly without a corresponding change in Secchi depth (Fig. 3), and cladocerans have increased significantly with weak declines in rotifers and copepods (Fig. 4). The MAR food web analysis results suggest that this community responds strongly to temperature, and that cladocerans and copepods interact differently with phytoplankton (Fig. 5). Thus, shifting zooplankton dominance could have community-wide implications. We discuss each of these results in greater detail and within their larger contexts as follows.

### Temperature

The ice-free season in Lake Baikal is known to have lengthened by 16.1 days over the past 137 years (Magnuson *et al.*, 2000) primarily related to later ice onset (Todd & Mackay, 2003). Annual mean air temperature in the Baikal area increased 1.2 °C over the past century, a rate twice that of the global average (Shimaraev *et al.*, 2002) and consistent with other reports of intense warming in higher latitudes (Serreze *et al.*, 2000). Recent studies in the Siberian Arctic indicate rapid changes in thaw lakes (Smith *et al.*, 2005; Walter *et al.*, 2006;) and river flow (Peterson *et al.*, 2002) that are associated with higher temperatures; however, studies in the Siberian subarctic are limited. The limnological dataset here reveals corresponding warming for subarctic Lake Baikal. Water temperature within the top 25 m in Lake Baikal increased over the past 60 years, with changes being most evident in the surface waters (0 m) during summer and fall (Fig. 2). Surface waters warmed at an average rate of approximately 0.20 °C decade^−1^, and the mean temperature at 25 m increased by 0.12 °C decade^−1^ over the 60-year period of observation. This trend was even more dramatic in data segregated by season, where surface waters in summer increased in temperature by 0.38 °C decade^−1^, and waters 25 m deep in the fall increased by 0.22 °C decade^−1^ on average. These rates of warming are similar to those reported for other lakes in the world [reviewed by Coats *et al.* (2006)]. For example, Lake Tahoe has warmed at a rate of 0.015 °C yr^−1^ (volume-weighted average) from 1970 to 2002 with greatest warming occurring in October and at depths of 0 and 10 m (Coats *et al.*, 2006). While some ice-free lakes in Europe are experiencing strong warming in winter (e.g. Straile *et al.*, 2003), ice insulates Lake Baikal's surface waters from ambient temperature changes in the winter and recent air temperature analyses indicate that the Baikalian region has primarily experienced summertime warming since the mid-1980s (Kouraev *et al.*, 2007). Even so, this summer warming of surface waters seems to have allowed the deeper waters (25 m) to warm significantly during the fall and winter. Such long-term, multi-season increases in water temperature may jeopardize endemic, cold-water stenotherms (e.g. the pelagic amphipod *Macrohectopus branickii* and the golymyanka) unable to adapt evolutionarily or behaviorally. Paleolimnological evidence from arctic and subarctic lakes demonstrates the particularly strong potential for climate warming to reorganize biological communities in historically cold lakes, through a lengthening of the growing season and associated limnological changes (Smol *et al.*, 2005; Mackay *et al.*, 2006;).

### Chlorophyll and water clarity

While chlorophyll *a* increased rapidly in the summer over the past quarter century (300% on average since 1979; Fig. 4), this increase in algal biomass has not yet caused a significant reduction in mean Secchi depth (Fig. 3) – the depth at which a standard white disk disappears from view. The increasing chlorophyll *a* trend is robust when the upper strata (0, 0–10, and 0–25 m) are analyzed independently (*R*^2^>0.30, *P*<0.01), diminishing the probability that the difference between Secchi and chlorophyll readings could be explained by increasing deep chlorophyll maxima. Typically, chlorophyll and Secchi measurements have strong correspondence (Carlson, 1977), but the potential for mismatch between Secchi and chlorophyll measurements has been illustrated in both theoretical (Lorenzen, 1980; Preisendorfer, 1986;) and empirical investigations (Edmondson, 1980; Megard *et al.*, 1980;), particularly at low chlorophyll concentrations such as those in Baikal. At low chlorophyll concentrations, other factors affecting light attenuation may have relatively high importance (Lorenzen, 1980; Megard *et al.*, 1980;); sporadic terrigenous influence in Lake Baikal's southern basin (Heim *et al.*, 2005) is one factor that may have contributed to variability in the clarity–chlorophyll relationship here. While there is general correspondence between chlorophyll and Secchi measurements in these data that can be observed in the right panel of Fig. 3, chlorophyll has only modest explanatory value for Secchi depth across the time series (e.g. *R*^2^=0.30) and this explanatory power varies greatly across months, opening up the possibility for the Secchi and chlorophyll *a* trends to differ as they do. This discordance between the long-term trends presented by the relatively constant Secchi readings and the increasing summer chlorophyll concentration (Fig. 3) in Lake Baikal highlights the importance of establishing monitoring for ‘early warning’ before a need for monitoring may be perceived visually. Although Lake Baikal is still ultra-oligotrophic, a tripling of algal biomass over the last quarter century is substantial.

Potential drivers of the summer increase in algal biomass include wind-induced mixing of cells or nutrients from deeper depths into upper waters, increased nutrient loading from the watershed, and warming effects on primary production and nutrient recycling. All potential drivers may interact with stratification changes that alter algal exposure to light. Increasing water column stability is implied by the increasing difference in temperature between upper and lower strata (Fig. 2); however, temperature measurements at finer depth intervals would be necessary to quantify long-term changes in water column stability that could affect nutrient and light availability for algae. Sediment cores do not suggest eutrophication effects offshore (Mackay *et al.*, 1998), and a recent study of phytoplankton across Lake Baikal suggested that temperature and stratification explained spatial patterns of phytoplankton abundance without invoking presumed nutrient gradients from river mouths to the open water (Fietz *et al.*, 2005). The increase in algal biomass reported here (Fig. 3), in conjunction with reports of an increase in surface water input over the last century (Shimaraev *et al.*, 2002) plus a projected increase in precipitation in this region over the next century (Prowse *et al.*, 2006), underscore the importance of future monitoring of external nutrient inputs from the watershed for detecting possible eutrophication. Increased precipitation may not only bring nutrients but also dissolved organic carbon that fuels productivity through microbial pathways (Yoshioka *et al.*, 2002). Multiple stressors may be impacting Lake Baikal and interacting in numerous ways, with impacts potentially varying across the broad and heterogeneous expanse of the lake (Mackay *et al.*, 1998; Fietz *et al.*, 2005;).

Thus far, the relationship of Lake Baikal's summer phytoplankton community with the long-term warming trend appears unlike that in Lake Tanganyika – a large-volume, tropical lake – where warmer water temperatures over much of the last century have reduced algal biomass and primary production indirectly via enhanced water column stability and reduced vertical mixing of nutrients (O'Reilly *et al.*, 2003; Verburg *et al.*, 2003;). In Baikal, the increasing temperature difference between surface and subsurface layers may ultimately result in lower nutrient availability in surface waters due to reduced mixing, but such an effect is not yet reflected in chlorophyll *a* or algal abundance. A larger temperature increase between surface and subsurface layers would be required to impede mixing in Lake Baikal than in tropical lakes such as Lake Tanganyika; water column stability increases less sharply with temperature in cold waters (7–15 °C summer Lake Baikal) than in warm waters (>20 °C, tropical lakes). In addition, water circulation in Lake Baikal differs from that of tropical Lake Tanganyika in a manner that may fundamentally affect the response of each to climate change; for example, deep water renewal in Lake Baikal appears to be the result of sporadic downwellings of surface waters, whereas Tanganyika's deep water layer is probably renewed from plunging riverine inputs (Wuest *et al.*, 2005). Warming in the Lake Baikal region may also have been accompanied by changes in wind and storm patterns that can affect water circulation patterns, phenomena that are currently under investigation.

Lake Baikal is unusual among the world's great lakes in harboring its highest algal biomass under ice (Fig. 3). Theoretical work (Kelley, 1997) suggests that this phenomenon has been dependent on clear, snow-free ice that allows enough light to fuel algal growth, and to warm near-surface water such that mixing caused by convection is sufficient to suspend the relatively heavy, nonmotile diatoms. In Baikal, under-ice blooms are frequently large endemic diatoms such as *Aulacoseira baicalensis* (Kozhova & Izmest'eva, 1998; Mackay *et al.*, 2006;). The prominence of under-ice algal blooms in Lake Baikal raises the possibility for dramatic restructuring of the community should patterns of snow fall or ice formation change over time (Mackay *et al.*, 2006) increasing or decreasing light availability to spring diatoms and exposing surface water to higher air temperature.

### Zooplankton community shifts

Baikal's longer ice-free season (Livingstone, 1999; Magnuson *et al.*, 2000;) and warmer temperatures during summer correspond with increasing prominence not only of the summer blooming algae but also unique members of the zooplankton community (Fig. 4). The slow-growing copepods show a weak long-term decline and the rotifers have decreased more strongly. The copepods' modest decline also appears to include greater structure in long-term trends not removed with the DSTFT (Fig. 4) that complicates interpretation of their long-term dynamics. However, cladocerans (*Bosmina* and *Daphnia*) have increased dramatically. Indeed, while the trend demonstrated by log-transformed cladoceran densities in Fig. 4 is visually modest in the context of the variability in the signal (*r*^2^=0.08), when untransformed it represents a 334% increase in average density since 1946. A recent analysis of a different, less extensive monitoring dataset from Lake Baikal reflects these general patterns in zooplankton abundance over the past quarter century (Afanasyeva & Shimaraev, 2006). While copepod and rotifer declines were primarily in the colder months, the cladoceran increase has been evident in the warmest months of summer and fall (Fig. 4). Results from MAR models reflect the cladocerans' strong correlation with higher temperatures (Fig. 5), with algal abundance providing no predictive power for cladoceran population growth. Crustacean zooplankton in large lakes respond strongly to temperature, potentially through both direct (e.g. metabolism) and indirect (e.g. food web) effects, to an extent that temperature may be more descriptive of crustacean abundance than are proxies for system productivity (Patalas, 1990; Patalas & Salki, 1992;). Correspondence of cladocerans with regions of warmer water in Lake Baikal has been demonstrated previously, for this plankton assemblage in which endemic species, such as the copepod *Epischura baicalensis*, are characteristically cold stenotherms (Melnik *et al.*, 1998, 2006). Overall, the MAR results (Fig. 5) indicated that biological interactions among Lake Baikal plankton were dwarfed by the temperature effects in this very cold lake, painting an image of a lake in which both phyto- and zooplankton respond dramatically to relatively small increases in temperature.

Cladocerans are especially well known for gaining dominance in warm conditions (Patalas & Salki, 1992; Gillooly & Dodson, 2000;), and they have unusual potential for changing food web dynamics, relative to other zooplankton groups (Sommer & Sommer, 2006), through at least three strong characteristics. First, cladocerans are preferred forage for zooplanktivorous freshwater fishes (Mayer, 1997) and facilitate energy transfer between primary productivity and higher trophic levels. Second, cladocerans have high grazing rates which can depress algal, bacterial, and competitor abundance (Carpenter *et al.*, 2001; Sommer & Sommer, 2006;); consequently, the abundance of large-bodied cladocerans seems to determine whether or not trophic cascades occur when fish abundance changes in lakes (Carpenter *et al.*, 2001; Sommer & Sommer, 2006;). Third, not only can cladocerans alter nutrient cycling through rapid removal of algal cells, but also through phosphorus retention that is substantially higher than that of copepods (Elser & Urabe, 1999; Sommer & Sommer, 2006;). This last impact of cladocerans on nutrient cycling could have particular importance in an oligotrophic system like Lake Baikal that has been historically dominated by copepods. Differential feeding ecology and sequestration of phosphorus means that copepods typically remove relatively large cells and excrete phosphorus that may be used by other cells, while cladocerans take a wide range of cells and do not ‘fertilize’ the remaining algal cells with phosphorus to the same degree (Elser & Urabe, 1999; Sommer & Sommer, 2006;). Such nutrient recycling differences between copepods and cladocerans were suggested by the results from the MAR modeling (Fig. 5), which show significant negative relationships of cladocerans with some algal taxa and significant positive relationships of copepods with several algal groups. Alterations to internal cycling of nutrients can be especially important in large deep lakes with long residence times (Tilzer & Serruya, 1990). These results taken together suggest hypotheses that could be directly tested in the field and laboratory. The highly significant long-term changes in zooplankton abundance (Fig. 4) reported here lie within a great deal of natural variation, and provide additional demonstration of the importance of long-term data collection for the detection of pattern in ecosystems of concern, to achieve the large number of data points necessary to discern pattern from noise.

### Broader context

This extraordinary long-term dataset from Lake Baikal demonstrates that the planktonic community has responded to environmental change over the past 60 years, as have aquatic systems worldwide (Scheffer *et al.*, 2001; Hays *et al.*, 2005;). The maintenance of this monitoring program has defied political and financial obstacles throughout its 60-year history and now clearly illustrates the value of dedicated monitoring, as the international scientific community debates the allocation of limited funds. For Lake Baikal, such impressive baseline monitoring will be of inestimable value as human activities, such as development of the watershed, proceed against the uncertain backdrop of climate change. Modern climate shifts in Russia – both figurative and literal – underscore the importance of increasing the international awareness of and access to these data from Lake Baikal, as Russia contemplates its scientific and environmental future and as Siberia warms.
